# Ascorbic acid pre-treated quartz stimulates TNF-α release in RAW 264.7 murine macrophages through ROS production and membrane lipid peroxidation

**DOI:** 10.1186/1465-9921-10-25

**Published:** 2009-03-19

**Authors:** Sonia Scarfì, Mirko Magnone, Chiara Ferraris, Marina Pozzolini, Federica Benvenuto, Umberto Benatti, Marco Giovine

**Affiliations:** 1 Department of Experimental Medicine, Section of Biochemistry, University of Genova, 16132 Genova, Italy; 2 Advanced Biotechnology Center, 16132 Genova, Italy; 3 Neuroimmunology Unit, Department of Neurosciences, Ophthalmology and Genetics and Center of Excellence for Biomedical Research, University of Genova, 16132 Genova, Italy; 4 Department of Biology, University of Genova, 16132 Genova, Italy

## Abstract

**Background:**

Inhalation of crystalline silica induces a pulmonary fibrotic degeneration called silicosis caused by the inability of alveolar macrophages to dissolve the crystalline structure of phagocytosed quartz particles. Ascorbic acid is capable of partially dissolving quartz crystals, leading to an increase of soluble silica concentration and to the generation of new radical sites on the quartz surface. The reaction is specific for the crystalline forms of silica. It has been already demonstrated an increased cytotoxicity and stronger induction of pro-inflammatory cyclooxygenase-2 (COX-2) by ascorbic acid pre-treated quartz (QA) compared to untreated quartz (Q) in the murine macrophage cell line RAW 264.7.

**Methods:**

Taking advantage of the enhanced macrophage response to QA as compared to Q particles, we investigated the first steps of cell activation and the contribution of early signals generated directly from the plasma membrane to the production of TNF-α, a cytokine that activates both inflammatory and fibrogenic pathways.

**Results:**

Here we demonstrate that TNF-α mRNA synthesis and protein secretion are significantly increased in RAW 264.7 macrophages challenged with QA as compared to Q particles, and that the enhanced response is due to an increase of intracellular ROS. Plasma membrane-particle contact, in the absence of phagocytosis, is sufficient to trigger TNF-α production through a mechanism involving membrane lipid peroxidation and this appears to be even more detrimental to macrophage survival than particle phagocytosis itself.

**Conclusion:**

Taken together these data suggest that an impairment of pulmonary macrophage phagocytosis, i.e. in the case of alcoholic subjects, could potentiate lung disease in silica-exposed individuals.

## Background

Inhalation of crystalline silica can induce a pulmonary inflammation characterized by the slow formation of lung fibrotic nodules that cause impairment of pulmonary function, eventually leading to death [1].

The severity of the pathology strictly depends on the structural characteristics of quartz particles. The dimension and composition of the dust and the chemical history of the solid matter from which the fractured silica derives, are a fundamental aspect for the generation of the disease [2,3].

It is commonly accepted that the disease originates from phagocytosis of quartz particles by alveolar macrophages: the inability of the cells to completely dissolve the crystalline structure then develops into a chronic inflammation [4].

Ascorbic acid (AA) has been shown to partially dissolve the quartz surface: the chemical erosion of the quartz releases soluble silica into the medium and generates radical sites on the quartz surface [5,6]. This reaction is specific for the crystalline forms of silica, the amorphous ones remaining unchanged [6,7]. These findings could be relevant to mammalian quartz toxicity as AA is present in the lung epithelium surfactant [8]: the surface modifications of the quartz crystals induced by AA may increase reactive oxygen species (ROS) production in the alveoli enhancing quartz fibrogenicity and carcinogenicity [1]. Indeed, the fact that AA specifically interacts with crystalline and not with amorphous silica could explain the requirement for crystalline silica for development of the fibrotic lesions [9].

We recently demonstrated increased particle cytotoxicity and induction of pro-inflammatory cyclooxygenase-2 (COX-2) by AA pre-treated quartz (QA) compared to untreated quartz (Q) in the murine macrophage cell line RAW 264.7, a cell model widely used [10,11].

Alveolar macrophages, activated by quartz particles, release fibrogenic factors and cytokines [12-14], among which TNF-α plays a crucial role as modulator of both the fibrogenic and inflammatory responses [15].

Macrophages exposed to silica *in vitro *release significant amounts of TNF-α [16,17], which in turn activates important pathways leading to the transcriptional up-regulation of fibrogenic proteins and TNF-α itself [18,19]. Mice exposed to silica and treated with an anti-TNF-α antibody show significantly reduced lung collagen deposition; conversely administration of recombinant TNF-α increases lung fibrosis [20]. Today, still little is known on the early macrophage response to silica particles, when the dust comes in contact with the plasma membrane through the scavenger receptors leading to phagocytosis.

We investigated the early events leading to release of TNF-α by quartz-stimulated murine RAW 264.7 macrophages, taking advantage of the increased macrophage response to AA-treated (QA) compared to untreated quartz (Q).

Results obtained indicate that lipid peroxides, generated directly from contact of the plasma membrane with quartz are sufficient to trigger a significant transcription and production of TNF-α, even in the absence of phagocytosis.

## Methods

### Materials

All reagents were acquired from SIGMA-ALDRICH (Milan, Italy), unless otherwise stated.

### Cell cultures

The mouse macrophage cell line RAW 264.7 was obtained from the American Type Culture Collection (Rockville, MD, USA). Rat alveolar macrophages were obtained by bronchoalveolar lavages (BAL) from healthy animals as described below. Cells were cultured at 37°C in a humidified, 5% CO_2 _atmosphere in D-MEM with glutamax (Lonza Milano srl, Milan, Italy), supplemented with 10% Defined Fetal Bovine Serum (HyClone, Logan, Utah, USA) (complete medium). Cell stimulation using different concentrations of sterilized quartz (MIN-U-SIL 5: US Silica, Berkeley Spring Plant, SSA _BET _= 5.2 m^2^/g) was obtained by adding 15, 50 or 100 μg/ml of distilled water- (Q) or AA-treated (QA) particles [prepared as described in 10, 7]. In detail, in terms of surface area/incubation volume 15, 50 and 100 μg/ml of MIN-U-SIL quartz particles corresponded to 0.75, 2.6 and 5.2 cm^2^/ml. Before use, sterilized quartz particles were assayed for the presence of endotoxin by using an end-point chromogenic assay (LAL Pyrochrome Kit, International PBI SpA, Milan, Italy) and following the manufacturer's instructions. Endotoxin content was always comprised between 0.005 and 0.01 EU/ml corresponding to 1–2 pg/ml of lipopolysaccharide (3 log below the lowest concentration commonly used on these cells to study its effects).

### Collection of alveolar macrophages from BAL samples

Male Sprague Dawley rats (8–10 weeks) were purchased from Harlan Italy (S. Pietro al Natisone, Italy) and housed at the animal facility of the Biochemistry Section in the Department of Experimental Medicine of the University of Genova. The program of animal use was approved by the CBA ethics committee, and all procedures involving animals were performed under protocols approved by the European Community directives.

Alveolar macrophages were obtained from bronchoalveolar lavages (BAL) as we have previously described [7]. Cells were then resuspended in complete medium, 1–1.5 × 10^6 ^cells/well (depending on the number of cells collected from each group) were seeded onto 60 × 15 mm tissue culture dishes (Falcon Becton Dickinson, Franklin Lakes, NJ, USA) and cultured for 6 hours at 37°C. The medium containing non-adherent cells was discarded, 100 μg/ml of Q or QA particles in complete medium was added to the cultures which were further incubated for 6 hours at 37°C. TNF-α production was quantified as described below.

### Measurement of murine TNF-α production

Production of murine TNF-α in RAW 264.7 macrophages after 6 or 18 hours incubation with 15, 50 or 100 μg/ml of AA-treated (QA) or untreated quartz (Q) in the presence or absence of 1 ng/ml murine Interferon-γ, in RAW 264.7 cells or in rat alveolar macrophages, was quantified in the culture medium. Untreated cultures were used as controls. Alternatively cells were pre-incubated in the presence or absence of 100 μg/ml dextran-sulphate (DXS) for 45 min, or 500 μg/ml butylated hydroxytoluene (BHT) for 15 min before adding 100 μg/ml Q or QA particles. Briefly, 3 × 10^6 ^cells were seeded onto 60 × 15 mm tissue culture dishes (Falcon BD) and cultured as described above; after 18 hours the stimuli were added to the culture media and cells were further incubated for 6 or 18 hours at 37°C. The culture media collected at 6 or 18 hours were centrifuged at 900 ***g ***for 5 min (Allegra X-22R centrifuge, Beckman Coulter SpA, Milan, Italy) and TNF-α concentration then quantified on the supernatants.

**Figure 1:**
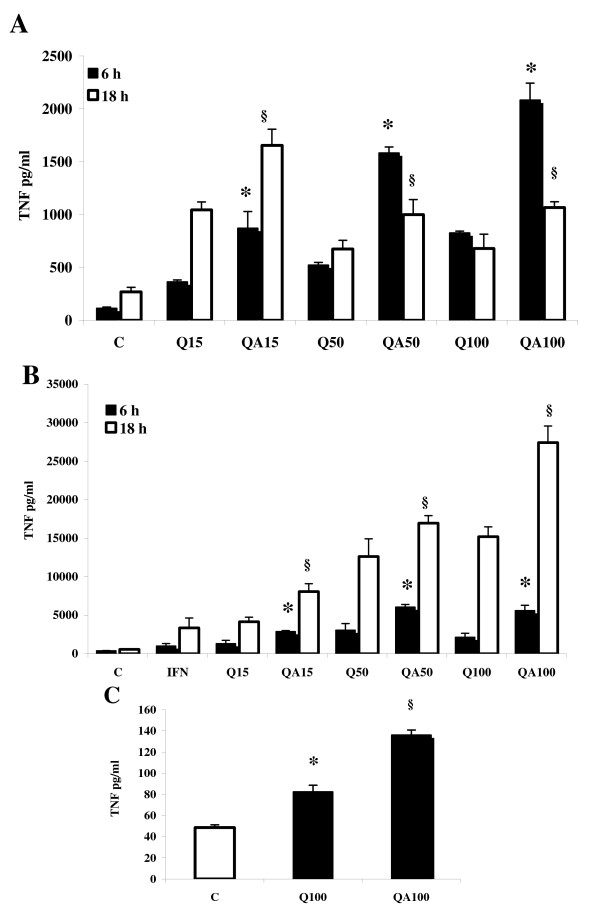
**TNF-α production in quartz-incubated RAW 264.7 cells**. (**A**) TNF-α concentration detected in RAW 264.7 cell supernatants after 6 hrs (black bars) and 18 hrs (white bars) stimulation with quartz, as determined using the L929 bio-assay. Cells were challenged with untreated (Q) or AA-treated (QA) quartz particles at 15, 50 and 100 μg/ml. Values are the mean ± SD from 4 experiments. The asterisks indicate a statistically significant difference between Q and QA values, at all particle concentrations at 6 h (T test, *p *< 0.0005). The symbols § indicate a statistically significant difference between Q and QA values at all particle concentrations, at 18 h (T test, *p *< 0.0005). (**B**) TNF-α concentration detected in RAW 264.7 cell supernatants after 6 h (black bars) and 18 hrs (white bars) stimulation with QA or Q particles in the presence of murine Interferon-γ (1 ng/ml). Values are the mean ± SD from 4 experiments. The asterisks indicate a statistically significant difference between Q and QA values at all particle concentrations, at 6 h (T test, *p *< 0.0005). The symbols § indicate a statistically significant difference between Q and QA values at all particle concentrations, at 18 h (T test, *p *< 0.0005). (**C**) TNF-α production in rat alveolar macrophages stimulated with 100 μg/ml QA or Q particles was evaluated after 6 h incubation. Values are the mean ± SD from 3 experiments. The asterisk indicates a statistically significant increase of TNF-α expression between Q100 and C samples (T Test, *p *< 0.0005), while the symbol § indicate a statistically significant difference between Q100 and QA100 TNF-α values (T Test, *p *< 0.0005).

Two procedures of TNF-α assay were followed, using a Mouse TNF-α (Mono/Poly) OptEIA kit (BD Biosciences, Pharmingen, San Diego, CA, USA) and a cytotoxicity-based assay using the murine fibroblast L929 cell line [21,22]. The two procedures were applied to the first TNF-α quantification in RAW 264.7 cell supernatants after incubation with 15, 50 and 100 μg/ml Q or QA particles (see Figure 1A) yielding quite comparable results, therefore for all the following quantifications only the second method was used.

In detail, TNF-α released by stimulated RAW 264.7 cells was assayed by measuring its apoptotic effect on the murine fibroblast cell line L929. Experiments were perfomed in quaduplicate on 96-well plates, target cells were seeded at a concentration of 3 × 10^4 ^cells/well; after 18 hours, the culture medium was removed and the RAW 264.7 cell culture supernatants, ten-fold diluted in fresh medium, were added to the wells together with 1 μg/ml actinomycin D. A standard curve of TNF-α toxicity was obtained in parallel by adding recombinant murine TNF-α at concentrations ranging from 25 to 800 pg/ml. After 24 hours culture, cell viability was determined with the MTT-reduction assay [23]. Briefly, 20 μl of a 5 mg/ml solution of MTT (3-(4,5-dimethylthiazol-2-yl) 2-5-diphenyl-tetrazolium bromide) were added to each well, the plates were further incubated for 90 min at 37°C, the supernatant was then carefully removed from each well and the blue formazan crystals were solubilized with 200 μl/well DMSO. The optical absorbance at 570 nm was determined with a Bio-Rad Microplate reader 450 (Bio-Rad Laboratories srl, Milan, Italy).

### Quantitative RT-PCR

Total RNA from RAW 264.7 macrophages was extracted using RNAeasy Mini Kit (Qiagen SpA, Milan, Italy) and RNase-Free DNase Set (Qiagen) according to the manufacturer's instructions, from a starting material of 2 × 10^6 ^RAW 264.7 cells grown onto 60 × 15 mm tissue culture dishes (Falcon BD) in the presence of 100 μg/ml Q or QA for 30 min, 3 h and 24 h. Total cell cDNA was synthesized from 1 μg RNA in the appropriate buffer containing 5 mM MgCl_2_, 40 U ribonuclease inhibitor (RN_ASE_OUT; Invitrogen), 10 mM dithiothreitol, and 200 U Superscript™ III (Invitrogen), at 50°C for 50 min. Complementary RNA was then removed using 1 μL *Escherichia coli *RNase H (Invitrogen) at 37 C for 20 min. The amount of TNF-α mRNA, normalized to the relative GAPDH control, was determined by real-time quantitative PCR using a Chromo 4 instrument (MJ Research, Bio-Rad). PCR was performed in a 20-μl volume nuclease-free water containing 10 μl 2 × master mix iQ SYBR Green^® ^(Bio-Rad), 0.2 μM each primer, and 0.5 μl cDNA or negative control. All samples were analyzed in triplicate. The following PCR conditions to analyze TNF-α mRNA were used: 10 min initial denaturation followed by 40 cycles of denaturation at 95°C for 15 s, annealing and elongation at 60°C for 60 s. The fluorescence was measured at the end of each elongation step. In order to generate a melting temperature curve a slow heating (1°C per sec) of the amplified product from 55°C to 92°C was performed. This curve served as a specificity control. The entire cycling process, including data analysis, was performed using the DNA ENGINE OPTICON^® ^2 REAL-TIME DETECTION SYSTEM Software program (2.03 version). The sequences of the GAPDH (M32599) primers were: 5'-TCTCCCTCACAATTTCCATCCCAG-3' (forward primer) and 5'-GGGTGCAGCGAACTTTATTGATGG-3' (reverse primer). The sequences of the TNF-α primers were 5'-GACGTGGAAGTGGCAGAAGAG-3' (forward primer) and 5'-TGCCACAAGCAGGAATGAGA-3' (reverse primer). To detect the PCR efficiency for each couple of primers, an amplification curve was performed, using four different dilutions of cDNA. Data analysis to detect the relative gene expression of TNF-α, using the cDNA from untreated cells as calibrator sample, was performed with the comparative threshold Ct method [24] via GENEX software for the iCycler iQ Real Time Detection System (Bio-Rad) [25].

### Scavengers treatment

TNF-α production in RAW 264.7 macrophages after stimulation with 100 μg/ml Q and QA particles was also assessed in the presence of 4000 U/ml catalase (Sigma), 50 mM mannitol (Sigma) and 2 mM desferoxamine (Sigma). Cells were cultured as described above and the scavengers were added together with the quartz particles for a total incubation time of 6 hors. Subsequently, culture media were assayed for TNF-α presence as described above.

### Confocal microscopy analyses

All images were obtained using a Leica TCS SL confocal microscope equipped with argon/He-Ne laser sources and a HCX PL APO CS 63.0 × 1.40 oil objective.

For imaging quartz phagocytosis in RAW murine macrophages, cells were seeded in 4-well Lab-Teck chamber slides (Nalge Nunc Int., Naperville, IL, USA) at 100,000 cells/well; the day after cells were incubated for 45 min in the presence or absence of 100 μg/ml DXS or 2 μg/ml Cytocalasin B and then challenged with 100 μg/ml quartz for 1 hour. Images of living cells (4 × digital zoom) were then acquired, in single stacks, in the phase contrast mode while quartz autofluorescence was detected with the 633 nm laser line in an emission range of 635–700 nm.

For imaging lipid peroxidation on RAW 264.7 cells, macrophages were seeded in 4-well Lab-Teck chamber slides at 100,000 cells/well. The day after cells were incubated with or without 100 μg/ml DXS for 45 min 37°C and then with the specific lipid peroxidation dye BODIPY 581/591 C11 (Molecular Probes, Invitrogen, Carlsbad, CA, USA) at 5 μM concentration in complete medium for 30 min at 37°C. Cells were then washed with HBSS medium and then challenged with or without 100 μg/ml quartz particles. Images (8 × digital zoom) were then immediately acquired during 35 min-time lapse experiments recording 1 image/min. An energy laser of 15% was applied to the 488 nm line while a 40% energy was used with the 543 nm line. The increase in green fluorescence was monitored in the 500–535 nm emission range, meanwhile the decrease in red emission was observed in the 580–620 nm range, in the same interval of time.

### Quantification of free soluble silicates in RAW 264.7 cells

3 × 10^6 ^RAW 264.7 cells were plated onto 60 × 15 mm tissue culture dishes and allowed to adhere for 4 hours at 37°C. Cells were then pre-incubated in the presence or absence of 100 μg/ml DXS or of 2 μg/ml Cytocalasin B for 45 min before stimulation with 100 μg/ml quartz for 6 or 24 hours at 37°C. At the end of the incubation cells were washed twice with ice-cold PBS to completely remove extracellular quartz and lysed with a cell scraper in 400 μl silica-free water (Carl Roth Gmbh & Co, Karlsrue, Germany). Each sample was briefly sonicated and ultracentrifuged in a TL100 (Beckman) for 1 hour at 100,000 ***g ***at 4°C. Silicates were then measured on 100 μl supernatant by a Silicate Kit (silicic acid) (Merck Sharp & Dohme SpA, Rome, Italy) based on a colorimetric method according to the manufacturer's instructions.

### Lipid peroxidation analysis

A quantitative analysis of lipid peroxidation on RAW 264.7 cells, challenged with 100 μg/ml Q or QA particles in the presence or absence of DXS or Cytocalasin B, was obtained by fluorimetric measurement using the specific dye BODIPY 581/591 C11. Experiments were performed in quadruplicate on 96-well plates. RAW 264.7 cells were plated at a density of 50,000 cells/well, allowed to adhere over night, and then incubated with 100 μg/ml DXS or 2 μg/ml Cytocalasin B, for 45 minutes at 37°C. Cells were then incubated for 30 min at 37°C with 5 μM BODIPY 581/591 dye. After incubation with dye, cells were washed with HBSS Solution, incubated at 37°C for 15 minutes and then challenged with 100 μg/ml Q or QA particles for 1 hour. The plates were finally read on a Fluostar Optima BMG (Labtechnologies Gmbh, Offemburg, Germany) using 485/520 excitation/emission wavelengths.

### ROS detection

Experiments were performed in quadruplicate on 96-well plates. RAW 264.7 cells were plated at a density of 50,000 cells/well, allowed to adhere over night, and then incubated with or without 100 μg/ml DXS or with 2 μg/ml Cytocalasin B, for 45 minutes at 37°C. Cells were then washed once with HBSS Solution and incubated for 30 min at 37°C with 10 μM 2',7'-dichloro-dihydro-fluorescein diacetate dye (Molecular Probes, Invitrogen). After incubation with dye, cells were washed with HBSS Solution, incubated at 37°C for 15 minutes and then challenged with 15, 50 or 100 μg/ml Q or QA particles for 1 hour. The plates were finally read on a Fluostar Optima BMG using 485/520 excitation/emission wavelengths.

### Cell viability/cytotoxicity

Experiments were performed in quadruplicate on 96-well plates. RAW 264.7 were plated at a density of 25,000 cells/well and allowed to adhere overnight. Cells were incubated with 100 μg/ml DXS for 45 minutes or 500 μg/ml BHT for 15 minutes at 37°C and then Q or QA particles were added at 100 μg/ml final concentration for 24 hours at 37°C.

Reference standard curves were assayed in parallel during each experiment and cell number was deduced by linear regression analysis. The SYTOX DNA-staining assay was performed to evaluate both cell viability and cell death as already described [10]. Briefly, for cell viability test, medium with debris and dead cells was removed from wells, then adherent cells were gently washed with PBS at 37°C and lysed with 100 mM Tris pH 7.4, 154 mM NaCl, 1 mM CaCl_2_, 0.5 mM MgCl_2_, 0.1% NP40. Cell DNA was then stained adding 1 μM SYTOX Green Nucleic^® ^Acid Stain (Molecular Probes, Invitrogen). Conversely, to estimate cell death, 1 μM SYTOX Green was added directly to the wells without washing in order to stain only DNA from dead cells. After incubation with dye at room temperature for 15 min, the plates were read on a Fluostar Optima microplate reader using 485/520 ex/em wavelengths.

The rate of apoptosis in the same set of experiments was assessed by FACS analysis of plasma membrane phosphatydilserine-positive cells. To perform the analysis 3 × 10^6 ^RAW 264.7 cells were plated onto 60 × 15 mm dishes in complete medium and allowed to adhere overnight. Cells were then incubated with 100 μg/ml DXS for 45 minutes or 500 μg/ml BHT for 15 minutes at 37°C and then Q or QA particles were added at 100 μg/ml final concentration for 24 hours at 37°C. At the end of the incubation cells were washed with ice-cold HBSS and gently detached from the plate with a cell scraper in 300 μl HBSS. A 100 μl aliquot of each sample was then incubated, for 20 min on ice and in the dark, in the presence or absence of 5 μg/ml of anti-phoshatidylserine Alexa-488 conjugate monoclonal antibody (Upstate Inc., Charlottesville, VA, USA). Cells were then washed with 5 ml ice-cold HBSS and centrifuged at 400 × ***g ***for 5 min and then resuspended in 500 μl HBSS and counterstained with propidium iodide (1 μg/ml final concentration). Flow cytometric analyses were performed by FACS Canto flow cytometer (BD Biosciences) and data were analysed by DIVA software.

### Statistical analyses

All parameters were tested by paired *t*-test. *p *values < 0.05 were considered significant.

## Results

### TNF-α release by QA- versus Q-stimulated macrophages

The TNF-α production in RAW 264.7 murine macrophages stimulated with 15–50–100 μg/ml of AA-treated (QA) or untreated (Q) Min-U-sil quartz was quantified in the cell supernatant after 6 and 18 hours of incubation, using the standard cytotoxicity-based assay on murine fibroblast L929 cell line [22].

The TNF-α concentration in the medium of cells incubated for 6 (Figure 1A, black bars) or 18 hours (white bars) was significantly increased by QA and Q treatment as compared to control cells (C). Samples obtained from cells challenged with Q particles showed no significant differences between values recorded at 18 hours as compared to values obtained at 6 hours, except for cells incubated with 15 μg/ml of Q particles, where TNF-α release at 18 hours was higher than that recorded at 6 hours.

TNF-α production in cell cultures challenged with QA particles was always significantly higher compared to that measured in cultures stimulated with Q particles (*p *< 0.0005), ranging from 2.4- to 3-fold increase at 6 hours and from 1.5- to 1.6-fold increase at 18 hours.

RAW cells were also challenged with Q and QA particles co-stimulated with 1 ng/ml of murine Interferon-γ (IFN-γ), a cytokine released by activated lymphocytes, mimicking in our model a later stage of inflammation, when lymphocytes infiltrate the lung tissue in large number and contribute to the development of a chronic inflammatory state. In the presence of IFN-γ, TNF-α release into the medium was significantly increased by treatment of cells with Q and QA, by the particle concentration and by the incubation time in a dose- and time-dependent fashion (Figure 1B). Specifically, co-stimulation of RAW 264.7 cells with Q particles together with IFN-γ induced a higher increase of TNF-α production in all samples ranging from 2.6- to 7.9-fold increase at 6 hours (black bars), and from 7.7- to 25.2-fold increase at 18 hours (white bars), compared to the corresponding values in the absence of IFN-γ (Figure 1A).

Similarly to what observed in cells stimulated with Q particles, TNF-α release from cells challenged with QA particles was significantly increased in the presence of IFN-γ, with an increase ranging from 2.7 to 11.6 at 6 hours and from 7.7 to 25.7 at 18 hours as compared to cells stimulated with quartz particles alone. Furthermore, TNF-α release from cells stimulated with increasing concentrations of QA particles for 6 and 18 hours, in the presence of IFN-γ was always significantly higher compared to values recorded in the media of cells challenged with Q particles in the same conditions (*p *< 0.0005), with values ranging from 2- to 2.6-fold increase at 6 hours and from 1.4- to 2.2-fold increase at 18 hours.

TNF-α production was also evaluated in primary cultures of rat alveolar macrophages (AM) isolated from broncho-alveolar lavage (BAL) of healthy animals and challenged for 6 hours with or without 100 μg/ml Q or QA particles. TNF-α release (Figure 1C) in the cell medium was significantly increased by incubation with Q (1.7-fold) or with QA particles (2.8-fold) compared to control, untreated cells (C) (*p *< 0.0005), however this increase was significantly lower as compared to the amount of TNF-α released by Q- or QA-stimulated RAW 264.7 cells at the same particle concentration. This difference could be due to various reasons: i) the number of AM recovered from BALs of rats did not allow to attain the same concentration of cells as in RAW 264.7 experiments (1–1.5 × 10^6 ^cells/well for AM treatments against 3 × 10^6^/well for RAW 264.7 treatments), and ii) it has already been reported the production of lower levels of TNF-α stimulated by concentrations of quartz similar to the ones used in our experiments in AM primary cultures as compared to other macrophage cell line cultures [11,26].

All the data obtained were further confirmed by use of monoclonal EIA Kit (see Material and Methods for details) yielding closely comparable results (not shown).

### Time-course of TNF-α mRNA synthesis in QA- versus Q-stimulated RAW 264.7 macrophages

**Figure 2:**
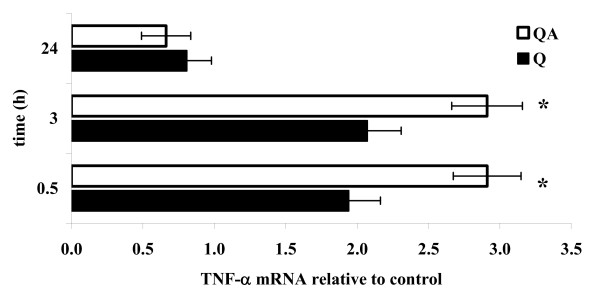
**RT-PCR of TNF-α mRNA in quartz-treated RAW 264.7 cells**. TNF-α mRNA transcription was monitored in RAW 264.7 macrophages by RT-PCR analysis from 0.5 to 24 h following cell stimulation with 100 μg/ml of QA (white bars) or Q particles (black bars). Results are the mean of three independent experiments performed in triplicate, and are expressed as TNF-α mRNA, normalized on the GAPDH transcription, relative to control cells at time-zero. The asterisks indicate a significant difference between samples challenged with Q and QA particles (T Test, *p *< 0.025).

TNF-α mRNA synthesis was measured by quantitative RT-PCR analysis in RAW 264.7 macrophages incubated with 100 μg/ml Q or QA particles for 30 min, 3 hours or 24 hours and compared to values recorded at the same time points on untreated, control cells (Figure 2).

TNF-α transcription was significantly induced 30 min and 3 hours after cell exposure to both Q (black bars) and QA particles (white bars), while for both stimuli TNF-α mRNA returned to basal, unstimulated values after 24 hours. QA particles induced a significant higher TNF-α transcription than Q particles (2.8-fold vs. 2.1-fold increase over controls, mean of values recorded at 30 min and 3 hours, QA vs Q *p *< 0.025).

### Role of oxygen radicals in quartz-induced TNF-α production

**Figure 3:**
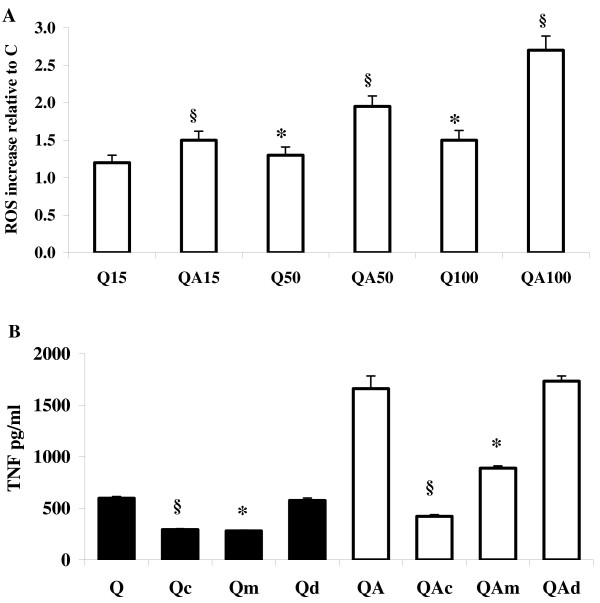
**ROS and TNF-α production in quartz-stimulated cells in the presence of ROS scavengers**. (**A**) Fluorimetric measurement of ROS production in RAW 264.7 macrophages challenged with 15, 50 and 100 μg/ml Q or QA particles. Values are the mean ± SD from 4 experiments. The symbol § indicates a statistically significant difference of QA-challenged cells compared to control, untreated cells (C), (T test, *p *< 0.0005); the asterisk indicates a statistically significant difference of Q-challenged cells compared to C (T test, *p *< 0.0005). (**B**) TNF-α production was evaluated in RAW 264.7 cells after 6 hrs incubation with 100 μg/ml Q or QA particles (black bars: Q samples; white bars: QA samples) in the presence of 4000 U/ml catalase (Qc, QAc bars), 50 mM mannitol (Qm, QAm bars) and 2 mM desferoxamine (Qd, QAd bars). Values are the mean ± SD from 4 experiments. The symbol § indicates a statistically significant difference between Q and Qc bars and between QA and QAc bars (T test, *p *< 0.0005). The asterisks indicate a statistically significant difference between Q and Qm, and between QA and QAm bars (T test, *p *< 0.0005).

ROS production was evaluated with a ROS-specific fluorescent probe after 1 hour incubation of RAW 264.7 cells in the presence of Q or QA particles (Figure 3A). For both stimuli ROS production was dose- and time-dependent, with QA inducing a significantly higher increase compared to Q particles at all concentrations tested. The most relevant differences were recorded at 100 μg/ml particles concentration with a 2.6-fold increase in ROS production in QA-stimulated cells as compared to control, untreated cells, and 1.8-fold increase as compared to Q-stimulated cells.

To verify whether the increased ROS generation induced by QA vs. Q particles was responsible for the increased TNF-α production observed with QA vs. Q particles, we explored the effect of ROS scavengers on TNF-α release by RAW 264.7 cells challenged with Q or QA. Cells were incubated for 6 hours with Q or QA particles in the presence or absence of an excess of the ROS scavengers catalase (H_2_O_2_), mannitol (OH^•^) or desferoxamine (Fe^2+ ^chelant) and TNF-α release was quantified in the cell supernatant. The three compounds alone were devoid of any effect on TNF-α production in these cells (not shown). TNF-α production, induced by Q and QA particles, was inhibited by catalase and mannitol, while desferoxamine showed no effect Figure 3B). In particular, catalase significantly inhibited (51%) TNF-α release induced by Q particles (Q versus Qc bar, *p *< 0.0005), the inhibition more effective in cells challenged with QA particles (75%; QA versus QAc bar, *p *< 0.0005).

Mannitol was similarly efficient in the inhibition of TNF-α production stimulated by Q and QA particles in RAW 264.7 macrophages. In particular, the cells showed a significant, 55% inhibition of TNF-α release when challenged with Q particles in the presence of mannitol (Q versus Qm bar, *p *< 0.0005), while in the presence of QA particles the mannitol-induced inhibition of TNF-α production reached 46% value (QA versus QAm bar, *p *< 0.0005).

Summarizing, catalase significantly reduced TNF-α release triggered by Q and QA particles, indicating a fundamental role of H_2_O_2 _in the signal transduction pathway leading to the cytokine production. The similar extent of inhibition exerted by mannitol on Q and QA-induced TNF-α production suggests a role also for OH^• ^radicals in stimulating protein synthesis. Conversely, the absence of any protective effect by desferoxamine (at the concentrations tested) suggests that redox reactions involving free iron, such as the Fenton reaction, are not involved in the eventual induction of TNF-α.

### The scavenger receptor antagonist dextran-sulphate inhibits quartz phagocytosis in RAW 264.7 cells

**Figure 4:**
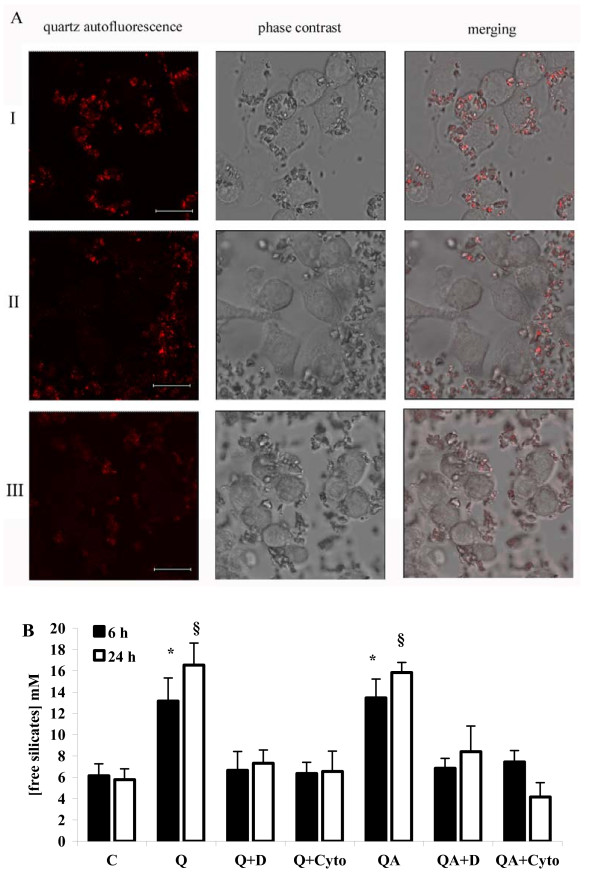
**Phagocytosis in quartz-stimulated cells in the presence of DXS and Cytocalasin B**. (**A**) Confocal microscopy analysis of quartz phagocytosis in RAW 264.7 cells (single stacks acquired with oil objective 63.0 ×, digital zoom 4 ×), red staining marking quartz autofluorescence. I, cells challenged for 1 h with 100 μg/ml quartz particles; II, the same in the presence of 100 μg/ml DXS, and III in the presence of 2 μg/ml Cytocalasin B. The white bar spans 8 μm. (**B**) Analysis of free intacellular silicates in RAW 264.7 cells challenged with or without (C) 100 μg/ml Q or QA in the presence (Q+D, QA+D and Q+Cyto, QA+Cyto) or absence of 100 μg/ml DXS or 2 μg/ml Cytocalasin B, after 6 h (black bars) or 24 h (white bars). Values are the mean ± SD from 4 experiments. The asterisk indicates a statistically significant difference between Q or QA and C at 6 h (T test, *p *< 0.0025), while the symbol § indicates a statistically significant difference between Q or QA and C at 24 h (T test, *p *< 0.0005).

It is known that macrophage scavenger receptors are the main route of internalization of Q particles in phagocytes [27]. In order to investigate whether the scavenger receptor activation triggered by contact with quartz particles is a necessary step to initiate quartz internalization in our cell model, RAW 264.7 cells were incubated with the scavenger receptor inhibitor DXS [28-30] or a well known inhibitor of phagocytosis, Cytocalasin B, at a concentration of 2 μg/ml, and then challenged with 100 μg/ml Q particles. Phagocytosis was then evaluated by confocal microscopy observation in time-course experiments while the concentration of intracellular soluble free silicates was quantified after 6 and 24 hours of incubation (Figure 4).

As shown in Figure 4A panel I, RAW 264.7 cells were able to internalize almost all surrounding Q particles, which could be easily observed within the cells after 1 hour incubation, due to Q auto-fluorescence (red staining). Conversely, co-incubation of RAW 264.7 cells with DXS or with Cytocalasin B (Figure 4A, panel II and III, respectively) significantly impaired Q phagocytosis; after 1 hour, the majority of Q particles being still on the coverglass surface.

The amount of intracellular free silicates in RAW 264.7 cells after internalization of Q and QA particles was quantified, in the presence or absence of DXS and of Cytocalasin B, by a colorimetric method. Surprisingly, the intracellular free silicates concentration in cells incubated with both Q and QA particles increased over values recorded in control, untreated cells indicating a partial erosion of the crystals (Figure 4B, two-fold after 6 h, Q and QA vs C, *p *< 0.0025; three-fold after 24 h, Q and QA vs C, *p *< 0.0005). Conversely, RAW 264.7 cells incubated with Q or QA particles in the presence of DXS or of Cytocalasin B (Q+D and Q+Cyto bars, respectively) showed no increase of the intracellular free silicates concentration as compared to control cells, confirming the inhibition of quartz phagocytosis by DXS and Cytocalasin observed by confocal microscopy (Figure 4A).

### Effect of DXS and Cytocalasin B on lipid peroxidation and ROS production in RAW 264.7 cells challenged with quartz

The observation by confocal microscopy that RAW 264.7 cells incubated with DXS and then challenged with Q particles were unable to phagocytose but retained the ability to establish a contact with crystalline silica, prompted us to explore the possibility that the first step of the macrophage response to quartz, i.e. when the plasma membrane interacts with the particles, is sufficient to trigger macrophage activation and TNF-α release.

We studied the effect of quartz on plasma membrane by evaluating the rate of lipid peroxidation on RAW 264.7 cells, challenged with Q and QA particles, in the presence or absence of DXS.

**Figure 5:**
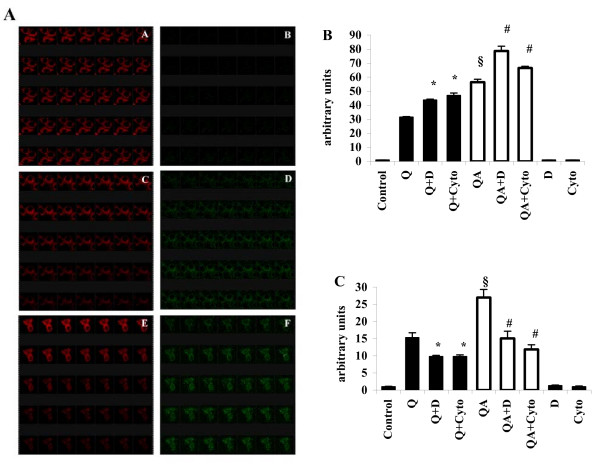
**Lipid peroxidation and ROS production in quartz-stimulated cells in the presence of DXS and Cytocalasin B**. (**A**) Confocal microscopy analysis of lipid peroxidation of RAW 264.7 cells (single stacks acquired with oil objective 63.0 ×, digital zoom 8 ×) using the specific fluorescent probe BODIPY 581/591 in time-lapse experiments, with acquisitions of 1 slide/min. A, C, E show the decrease of red fluorescence during 35 min, while B, D, F show the contemporary increase of green fluorescence in the same cells. A, B control, untreated cells; C, D cells challenged with 100 μg/ml quartz particles; E, F cells challenged with quartz particles in the presence of 100 μg/ml DXS. (**B**) Fluorimetric quantitation of lipid peroxidation in RAW 264.7 cells challenged with 100 μg/ml Q or QA (white bars) particles in the presence (Q+D, QA+D and Q+Cyto, QA+Cyto) or absence of 100 μg/ml DXS or of 2 μg/ml Cytocalasin B for 1 h. Values are the mean ± SD from 4 experiments. The symbol § indicates a statistically significant difference between Q and QA challenged cells (T test, *p *< 0.0005); the asterisk indicates a statistically significant difference between Q+D or Q+Cyto and Q-challenged cells (T test, *p *< 0.005), while the symbol # indicates a statistically significant difference between QA+D or QA+Cyto and QA-challenged cells (T test, *p *< 0.005). (**C**) Fluorimetric measurement of ROS production in RAW 264.7 macrophages in the same conditions of (B) after 1 h incubation. Values are the mean ± SD from 4 experiments. The symbol § indicates a statistically significant difference between Q and QA challenged cells (T test, *p *< 0.0005); the asterisk indicates a statistically significant difference between Q+D or Q+Cyto and Q challenged cells (T test, *p *< 0.0025), while the symbol # indicates a statistically significant difference between QA+D or QA+Cyto and QA-challenged cells (T test, *p *< 0.0005).

Qualitative confocal microscopy studies were performed using a membrane intercalating fluorescent probe, BODIPY 581/591 C11 [31-33], which reveals lipid peroxidation through a specific fluorescence emission shift from red to green. Figure 5A shows the result of time-course experiments in which probe-loaded RAW 264.7 macrophages were monitored for lipid peroxidation in the presence of various stimuli. Control, untreated cells (panels A-B) showed no fluorescence shift during an observation time of 35 min (1 slide/min), while a clear shift from red to green was observed when cells were challenged either with quartz alone (panels C-D) or with quartz in the presence of DXS (panels E-F). Fluorimetric measurment of the green fluorescence of the same samples, allowed a quantification of the lipid peroxidation rate under these conditions. Results are shown in Figure 5B. As expected Q and QA particles increased lipid peroxidation in RAW 264.7 macrophages as compared to untreated, control cells, with QA showing significantly higher values than Q (*p *< 0.0005). The peroxidation values measured on RAW 264.7 cells incubated with DXS or Cytocalasin B, in the presence of Q (Q+D and Q+Cyto bars, respectively) or QA particles (QA+D and QA+Cyto bars) showed a further increase as compared to Q and QA particles alone (Q+D vs Q, *p *< 0.005; QA+D vs QA, *p *< 0.0005).

In parallel to the estimation of membrane lipid peroxidation, ROS production was measured in RAW 264.7 cells after 1 hour of incubation with Q and QA in the presence or absence of DXS and of Cytocalasin B. Along with lipid peroxidation ROS production was also increased in cells challenged with Q and, particularly QA particles (Figure 3A and Figure 5C). In contrast to lipid peroxidation, which increased in the presence of DXS and Cytocalasin B, ROS production in RAW 264.7 cells incubated with either Q or QA particles was significantly reduced both in the presence of DXS and of Cytocalasin B(Q+D and Q+Cyto vs Q, *p *< 0.0025; QA+D and QA+Cyto vs QA, *p *< 0.0005), in line with inhibition of phagocytosis by DXS and Cytocalasin B (Figure 4A) and of the subsequent respiratory burst.

### Effect of DXS and BHT on TNF-α production and cell viability in RAW 264.7 cells stimulated with quartz

The effect on TNF-α production and cell viability of the lipid peroxidation triggered by cell membrane-quartz particle contact was investigated.

**Figure 6:**
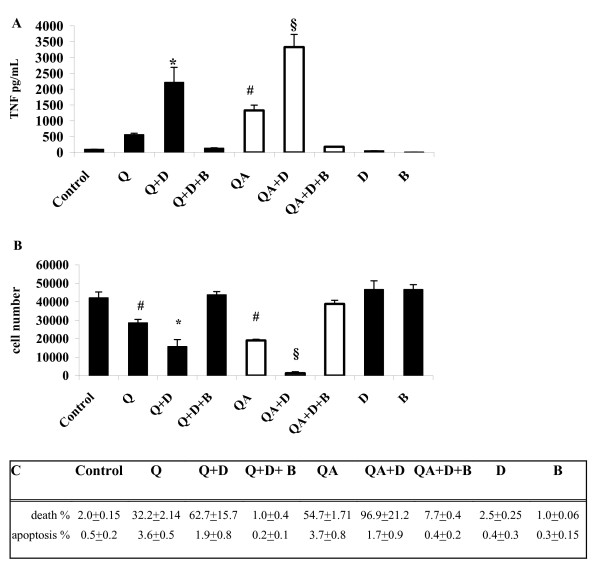
**TNF-α production and cell viability in quartz-stimulated cells in the presence of DXS**. (**A**) TNF-α production in cell supernatants after 6 h incubation with 100 μg/ml Q or QA (white bars) particles in the presence or absence of 100 μg/ml DXS (Q+D, QA+D), and of 500 μg/ml BHT (Q+D+B, QA+D+B). Values are the mean ± SD from 4 experiments. The symbol # indicates a statistically significant difference between Q and QA challenged cells (T test, *p *< 0.0005); the asterisk indicates a statistically significant difference between Q and Q+D challenged cells (T test, *p *< 0.0005), while the symbol § indicates a statistically significant difference between QA and QA+D treated cells (T test, *p *< 0.0005). (**B**) RAW 264.7 cell viability after 24 h incubation with 100 μg/ml Q or QA (white bars) particles in the presence or absence of 100 μg/ml DXS (Q+D, QA+D), and of 500 μg/ml BHT (Q+D+B, QA+D+B). Values are the mean ± SD from 4 experiments. The symbol # indicates a statistically significant difference between control, untreated cells (C) and Q and between C and QA (T test, *p *< 0.0005); the asterisk indicates a statistically significant difference between Q and Q+D challenged cells (T test, *p *< 0.0005), while the symbol § indicates a statistically significant difference between QA and QA+D treated cells (T test, *p *< 0.0005). (**C**) Percentage of RAW 264.7 cell death (measured by Sytox Green DNA assay, see Methods) and apoptosis (measured by FACS analysis of phosphatidylserine-positive cells, see Methods) after 24 h incubation with 100 μg/ml Q or QA particles in the presence or absence of 100 μg/ml DXS (Q+D, QA+D), and of 500 μg/ml BHT (Q+D+B, QA+D+B). Values are the mean ± SD from 3 experiments.

RAW 264.7 macrophages were incubated with Q or QA particles in the absence (controls) or in the presence of the scavenger receptor inhibitor DXS or of the lipid peroxidation scavenger BHT [34-36]. TNF-α production was quantified after 6 hours in the cell supernatant and cell viability, as well as apoptosis, was evaluated at 24 hours. When quartz particle phagocytosis was inhibited by DXS, TNF-α production was significantly increased in cells stimulated with either Q or QA particles (3.9- and 2.5-fold, respectively), over TNF-α release from cells challenged with Q or QA in the absence of DXS (Figure 6A, Q+D vs Q, *p *< 0.0005; QA+D vs QA, *p *< 0.0005). The stimulation of TNF-α release observed in Q- or QA-treated cells in the presence of DXS was prevented by BHT, indicating a causal role of lipid peroxides in the cytokine synthesis (Q+D+B and QA+D+B bars).

In line with a higher TNF-α production driven by the sustained membrane lipid peroxidation in RAW 264.7 cells stimulated with Q or QA particles in the presence of DXS, cell viability was also significantly affected. After 24 hours cell viability was reduced by 30% in cells incubated with Q particles and by 70% in cells challenged with QA particles compared to control, untreated cells (Figure 6B, Q and QA bars versus C). Cell viability was further reduced in cells co-incubated with DXS and Q particles, a 30% less as compared to cells incubated with Q alone (Q+D vs Q, *p *< 0.0005), while 97% cell death was observed in RAW 264.7 macrophages incubated with DXS and QA particles (QA+D vs QA, *P *< 0.0005). Pre-incubation of cells with the antioxidant BHT completely preserved cell viability in the presence of Q or QA particles co-incubated with DXS (Figure 6B, Q+D+B and QA+D+B bars).

The percentage of apoptotic cells was evaluated in parallel in the same set of experiments (Figure 6C). FACS analyses of phosphatydilserine-positive plasma membranes in cells challenged with Q or QA particles revealed a very low extent of apoptosis (3.6–3.7%) for both treatments. Necrosis, in turn, amounted to 32% in Q-treated cells and 55% in QA-treated cells after 24 hours. DXS-pre-treatment before addition of Q or QA resulted in even a higher level of necrosis with no significant variations of apoptosis compared to Q and QA treatments alone. Finally, BHT pre-incubation of cells before addition of particles in the presence of DXS, completely prevented both necrosis and apoptosis.

## Discussion

The toxicity of crystalline silica in mammals is known since many years to the medical community involved in the field of occupational diseases [1]. Development of a long-term lung disease upon inhalation of silica particles is known to correlate with silica properties such as the crystalline structure and the presence of freshly fractured particle surfaces [2,3,37,38]. Cell response to quartz particles appears to be strictly related to the abundance of surface radicals, which trigger macrophage activation by means of specific inflammatory and pro-apoptotic pathways [39].

Our group recently demonstrated that specific chemical modifications occurring on the quartz surface after exposure to ascorbic acid (AA) increase quartz cytotoxicity and macrophage activation, evaluated through the expression of cyclooxygenase-2 and production of prostaglandins in the murine cell line RAW 264.7 [7,10].

It is well known the presence of micromolar concentrations of AA in the lung fluids, where this molecule is believed to give a prominent contribution to the antioxidant defense [8]. Conversely, our findings are suggesting a new, peculiar role for AA in the pathogenesis of quartz-induced lung disease. The ability of AA to enhance the cytotoxic and pro-inflammatory properties of quartz particles in the lung may explain the specific reactivity of mammals towards quartz among the different structural forms of silica.

Results from other research groups have already put in evidence an involvement of AA in the development of quartz-induced lung injury *in vivo*, with the observation that guinea pigs fed an ascorbate-rich diet and exposed to quartz show a higher degree of infiltration of inflammatory cells in lung fluids compared to controls fed low doses of AA [40,41].

Taking advantage from the ability of AA to enhance macrophage response to quartz particles, we investigated the early events following quartz contact with the macrophage plasma membrane and triggering the synthesis and release of TNF-α, a key cytokine in the development of lung fibrosis after silica inhalation [20].

Results obtained demonstrate that TNF-α secretion and mRNA synthesis are significantly increased in RAW 264.7 macrophages challenged with AA-treated quartz (QA) compared to untreated quartz (Q) (Figure 1 and Figure 2), and that the higher pro-inflammatory effect of QA over Q is even more evident in the presence of IFN-γ (Figure 1b). This cytokine is produced by activated lymphocytes recruited by macrophages at a later stage of the inflammatory process [4]. These results suggest that the quartz surface modifications caused by AA could be relevant not only during the first steps of the lung inflammatory response but also subsequently. The long-lasting presence of quartz particles in the lung and their continuous exposure to AA, inducing the chemical modifications already demonstrated *in vitro *[6,10] may indeed favour an escalation of the inflammatory response, as observed during the development of silicosis [42].

Intracellular ROS play a fundamental role in the silica-induced transduction pathway leading to TNF-α production [43,44]. In our experimental model, both H_2_O_2 _and OH^• ^radicals were found to be key signals in the enhanced production of TNF-α in QA-stimulated cells (Figure 3), as cytokine synthesis was significantly reduced by presence of the two specific ROS scavengers catalase and mannitol. Conversely, the divalent-iron chelator desferoxamine, had no effect, suggesting a mechanism different from the Fenton reaction for generation of OH^• ^radicals. In fact, *in vitro *experiments have already demonstrated the generation of OH^• ^radicals from the direct reaction of H_2_O_2 _with the quartz surface, a mechanism strongly enhanced by the previous AA treatment of the crystalline dust [6,7,10,45].

To assess the direct role of the plasma membrane in the silica-induced TNF-α production, we focused on the very first step of macrophage phagocytosis of quartz which is known to be mediated by scavenger receptors [27,46,47]. Thus, we used a specific scavenger receptor inhibitor, dextran-sulphate (DXS), to impair phagocytosis of Q and QA particles in RAW 264.7 cells and establish the role of the contact between particle and plasma membrane in triggering TNF-α synthesis.

Pre-incubation with DXS, as well as with Cytocalasin B, a common phagocytosis inhibitor, completely inhibited quartz phagocytosis in RAW 264.7 cells as demonstrated by confocal microscopy images showing an absence of quartz internalization (Figure 4A, lane II and III).

Phagocytosis of quartz particles by RAW 264.7 cells was followed by a significant (three-fold) increase of the intracellular concentration of free silicates (Figure 4B), indicating that a partial dissolution of quartz indeed occurs after internalization, in contrast to the currently held inability of macropahges to dissolve quartz [1-4].

DXS treatment of the cells, and Cytocalsin B as well, also prevented the increase of the intracellular silica concentration, in line with its inhibitory effect on phagocytosis. DXS treatment prevented quartz phagocytosis and partial digestion by macropahges, but not the ability of cells to establish contact with the particles, as observed by confocal microscopy on living RAW 264.7 cells (Figure 4A). Membrane lipid peroxidation was significantly higher in QA- compared to Q-treated cells, and DXS pre-treatment further increased lipid peroxidation in both cell samples (Figure 5A and Figure 5B). These results, confirmed also by use of Cytocalsin B, another phagocytosis inhibitor, indicate that contact of quartz particles with the plasma membrane, which is prolonged in the absence of quartz internalization, is responsible for lipid peroxidation.

Conversely, pre-treatment of RAW 264.7 cells with DXS prior to challenge with Q and QA particles, significantly reduced ROS production which is secondary to phagocytosis.

Pointedly, in DXS-pretreated cells production of TNF-α increased signficantly after exposure to Q, and even more to QA, compared to cytokine release from cells where phagocytosis was not inhibited (Figure 6A). This result indicate that membrane lipid peroxides generated independently of phagocytosis induce production of TNF-α. This conclusion is further sustained by the complete inhibition of both Q- and QA-induced TNF-α synthesis in the presence of DXS by the lipid peroxide scavenger BHT. Both the two mechanisms, i.e. ROS production after quartz internalization and lipid peroxides generation during membrane-particle contact, are likely to happen physiologically and consequently being synergistic in activating macrophage response.

In line with the increased oxidative damage of the plasma membrane, viability of DXS-pretreated cells was severely reduced after 24 hours incubation with Q and, even more, with QA (Figure 6B and Figure 6C). The observed reduced cell viability was almost due to necrosis while the extent of apoptosis was minoritary (Figure 6C). The lipid peroxidation inhibitor BHT prevented cell death in cells challenged with Q or QA in the presence of DXS.

In conclusion, this study demonstrates that reaction of quartz with AA increases toxicity of crystalline silica inducing a higher macrophage production of TNF-α, the pro-inflammatory cytokine currently believed to be mainly responsible for generation of lung fibrosis after exposure to silica [20]. Furthermore, this is the first demonstration that plasma membrane contact with quartz, in the absence of phagocytosis, is sufficient to trigger membrane lipid peroxidation, TNF-α release and cell death and this appears to be even more detrimental to macrophage survival than particle phagocytosis itself.

Taken together these data suggest that an impairment of macrophage phagocytosis could exacerbate lung disease in silica-exposed individuals. This could be the case in alcoholic subjects, where pulmonary macrophage function is compromised, resulting in defective phagocytosis [48]. This would imply that alcohol abuse or other pathologic conditions impairing macrophage phagocytosis could be a risk factor for silica-induced lung disease and a detrimental condition facilitating complications by means of the prolonged macrophage contact with unphagocytosed particles.

## Abbreviations

AA: ascorbic acid; Q: untreated quartz; QA: ascorbic acid-pretreated quartz; ROS: reactive oxygen species; COX-2: cyclooxygenase-2; TNF: tumor necrosis factor; BAL: broncho-alveolar lavage; DXS: dextran sulphate; Cyto: Cytocalasin B; BHT: butyl hydroxytoluene; MTT: dimethylthiazol diphenyl-tetrazolium bromide; HBSS: Hank's balanced salt solution.

## Competing interests

The authors declare that they have no competing interests.

## Authors' contributions

SS: conception and design, acquisition of data, analysis and interpretation, manuscript writing; MM: conception and design, acquisition of data, analysis and interpretation; CF: acquisition of data, analysis and interpretation; MP: acquisition of data, analysis and interpretation; FB acquisition of data, analysis and interpretation, UB: critical revision and final approval; MG conception and design, critical revision and final approval.
